# Redirecting Primary Metabolism to Boost Production of Tyrosine-Derived Specialised Metabolites *in Planta*

**DOI:** 10.1038/s41598-018-33742-y

**Published:** 2018-11-22

**Authors:** Alfonso Timoneda, Hester Sheehan, Tao Feng, Samuel Lopez-Nieves, Hiroshi A. Maeda, Samuel Brockington

**Affiliations:** 1 Department of Plant Sciences, University of Cambridge, Cambridge, UK; 2 Department of Botany, University of Wisconsin–Madison, Madison, Wisconsin USA

## Abstract

*L*-Tyrosine-derived specialized metabolites perform many important functions in plants, and have valuable applications in human health and nutrition. A necessary step in the overproduction of specialised tyrosine-derived metabolites *in planta* is the manipulation of primary metabolism to enhance the availability of tyrosine. Here, we utilise a naturally occurring de-regulated isoform of the key enzyme, arogenate dehydrogenase, to re-engineer the interface of primary and specialised metabolism, to boost the production of tyrosine-derived pigments in a heterologous plant host. Through manipulation of tyrosine availability, we report a 7-fold increase in the production of tyrosine-derived betalain pigments, with an upper range of 855 mg·kg^−1^·FW, which compare favourably to many *in vitro* and commercial sources of betalain pigments. Since the most common plant pathway for tyrosine synthesis occurs via arogenate, the de-regulated arogenate dehydrogenase isoform is a promising route for enhanced production of tyrosine-derived pharmaceuticals in diverse plant hosts.

## Introduction

*L*-Tyrosine (Tyr) is an essential aromatic amino acid required for protein biosynthesis in all organisms, and is synthesised *de novo* in bacteria, fungi and plants, but not in animals. In addition to protein synthesis, plants utilise Tyr to produce a diverse array of specialized metabolites, which also have important applications as pharmaceuticals (e.g. epinephrine, noradrenaline, apomorphine, and morphinans)^1^. A well-established approach to isolate and overproduce Tyr-derived pharmaceuticals is reconstitution of the biosynthetic pathway in heterologous organisms, either in microbial or plant platforms, each of which have advantages and disadvantages^2^. Microbial hosts can be genetically manipulated in a rapid fashion, are fast growing, and have a well-established industrial-scale production infrastructure^2^. Plants, on the other hand, photosynthesise and do not require exogenous carbon sources, generate large amounts of biomass relatively cheaply, and offer a stable and scalable system, but are a less developed platform for metabolic engineering^2^.

Tyr is synthesised from prephenate, which is converted from chorismate, the final product of the shikimate pathway^3,4^. However, in the synthesis of Tyr, prephenate is processed differently in microbes versus plants. In most microbes, prephenate is oxidatively decarboxylated by prephenate dehydrogenase (TyrA_p_/PDH) to 4-hydroxyphenylpyruvate (HPP), which is transaminated to Tyr^5^. However, most plants first transaminate prephenate into arogenate and subsequently decarboxylate arogenate into Tyr by arogenate dehydrogenase (TyrA_a_/ADH)^6^, with both steps occurring in the plastids^7^. In plants, the Tyr pathway is usually highly regulated at ADH, which is strongly feedback inhibited by Tyr^6,8^. Consequently, if this negative feedback loop can be modulated, ADH offers a prospective gateway for the potential overproduction of Tyr-derived metabolites in plant hosts^9^. The production of Tyr-derived plant metabolites has already been achieved in microbial hosts, and considerable progress has been made in overriding the metabolic regulation of Tyr in these microbial platforms^10^. However, to our knowledge, mechanisms for overriding the metabolic regulation of Tyr in plant host platforms coupled to over-production of Tyr-derived metabolites have not yet been demonstrated.

**Figure 1:**
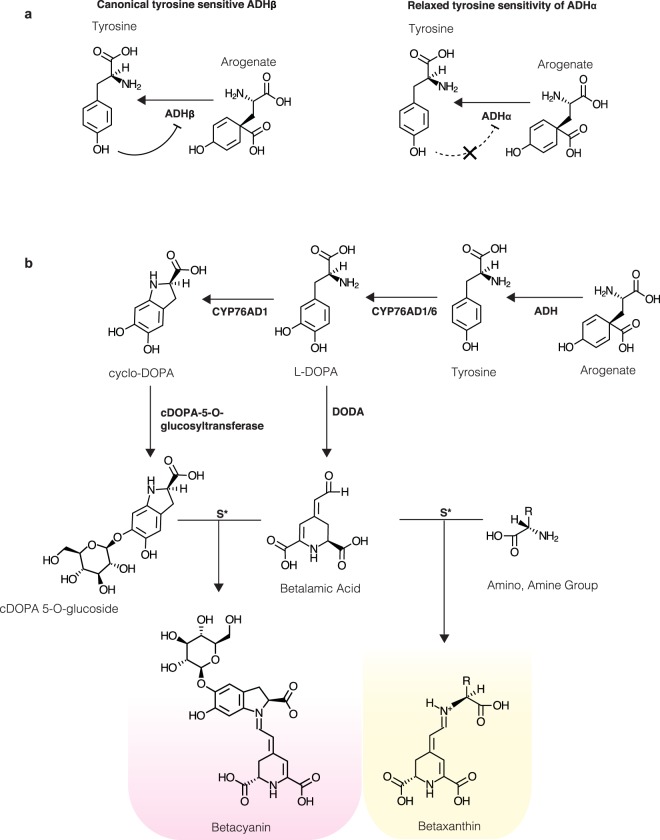
The tyrosine and betalain biosynthetic pathways. (**a**) The negative feedback loop exerted by tyrosine on the canonical ADHβ isoform, versus the relaxed tyrosine sensitivity of the ADHα isoform. (**b**) The first step shown is the step of arogenate decarboxylation catalysed by arogenate dehydrogenase (ADH) that is the focus of the current study. Subsequent illustrated betalain-committed steps include tyrosine 3′ hydroxylation catalysed by cytochrome P450 enzymes (CYP76AD1/AD6) to form *L*-DOPA, the formation of betalamic acid from *L*-DOPA through the action of 4,5 DOPA dioxygenase (DODA), the conversion of *L*-DOPA to cyclo-DOPA by cytochrome P450 (CYP76AD1), glucosylation of cyclo-DOPA via the action of cyclo-DOPA 5-O glucosyltransferase, and the spontaneous condensation of betalamic acid with cDOPA 5-O-glucoside to form purple betacyanins, or with amino or amine groups to form yellow betaxanthins.

We recently identified an isoform of the ADH enzyme (ADHα), that has relaxed sensitivity to the negative feedback inhibition by Tyr, and is implicated in the evolution of Tyr-derived pigments, betalains, in Caryophyllales (Fig. 1a)^8^. ADHα is the product of a gene duplication event specific to Caryophyllales, and most species in Caryophyllales, therefore, possess at least two copies of ADH – the de-regulated ADHα and a Tyr-sensitive ADHβ^8^. Clearly, the expression of the de-regulated ADHα isoforms in heterologous plant platforms has the potential to enhance the production of Tyr-derived metabolites. Betalains are one such example of Tyr-derived metabolites, and are a class of pigments comprising yellow-orange betaxanthins and red-violet betacyanins, unique to Caryophyllales^11,12^. Betacyanins are the major natural red-colour dyes used in the US food industry^11,12^. The committed biosynthetic pathway for betalains is relatively short, proceeds in three enzyme-mediated steps (Fig. 1b), and has been successfully expressed in both plant and microbial host platforms^13–15^. Betalain pigments have been previously employed as an enzyme-coupled bio-sensor to explore flux in the production of Tyr-derived metabolites^13^, offering a powerful tool to visualise the impact of manipulating primary Tyr metabolism. Here, we demonstrate the capacity of de-regulated ADH to, in turn, boost the production of Tyr-derived metabolites in the heterologous plant platform, *Nicotiana benthamiana*.

## Results and Discussion

**Figure 2:**
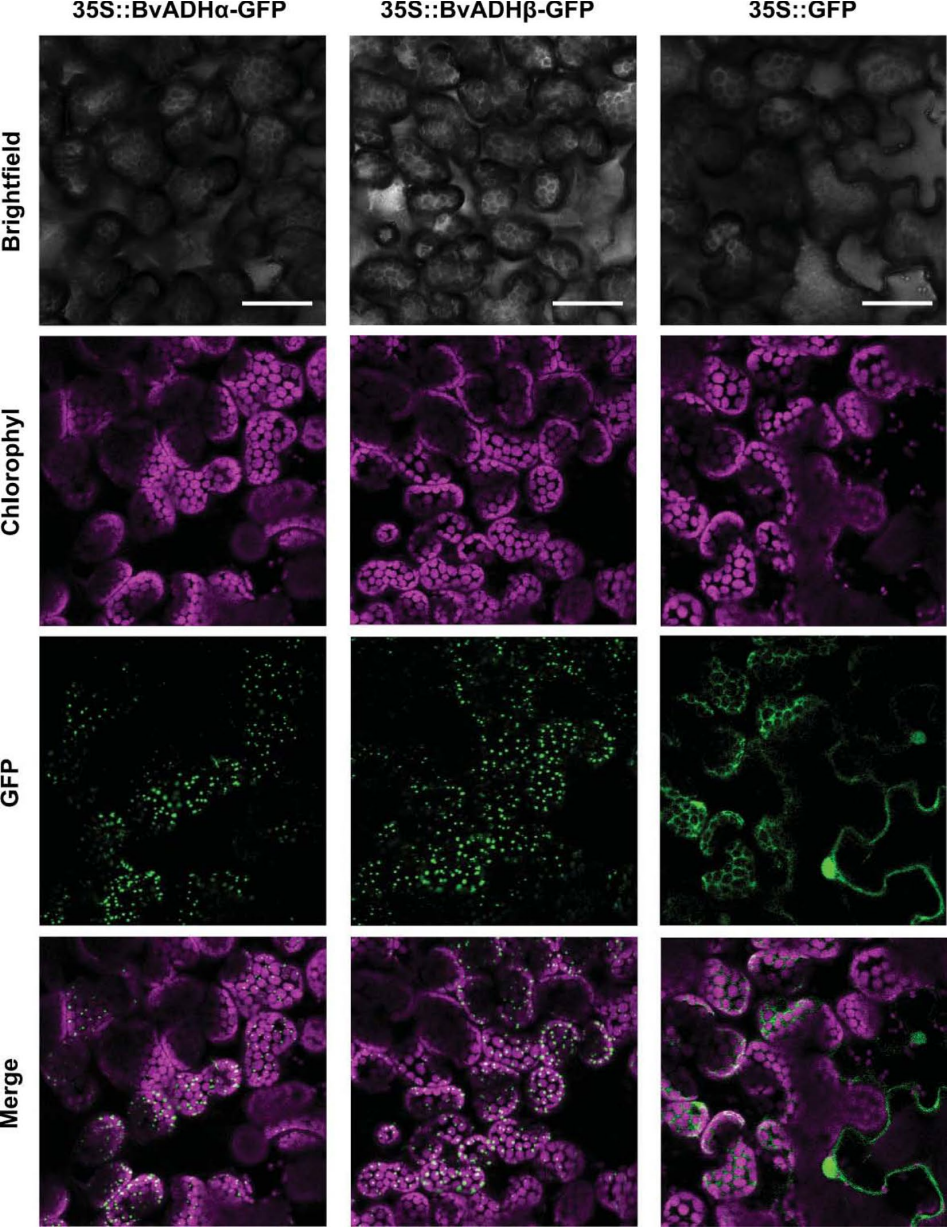
BvADH enzymes are targeted to the plastids *in planta*. Green fluorescence protein (GFP) was fused at the C-terminal end of BvADHα (left) and BvADHβ (middle) and transiently expressed in *N. benthamiana* leaves. Free GFP (right) was used as control for cytosolic localization. Representative images show GFP fluorescence and chlorophyll autofluorescence in green and purple, respectively. Merge (bottom) is a composite with GFP fluorescence and chlorophyll autofluorescence. Scale bars = 50 μm.

In our previous work, transient expression of a de-regulated ADHα driven by the CaMV 35S promoter resulted in an approximate ten-fold increase relative to its paralog, the Tyr-sensitive ADHβ^8^. While transient over-expression of the Tyr-sensitive ADHβ alone does not significantly enhance the production of *L*-Tyr (relative to a GFP expression control), the over-expression of the de-regulated ADHα increases Tyr levels by an order of magnitude^8^. We here examined the subcellular localization of these two paralogs following transient expression in *Nicotiana benthamiana* utilizing fusion of GFP to the C-terminus of the ADH isoforms. Consistent with previously observed plastid localization of the ADHα and ADHβ proteins in protoplast transformations^8^, we found in whole tissue visualization that both paralogs exhibit similar patterns associated with discrete patches at the plastids^16^ (Fig. 2). The location of both paralogs to the plastids emphasizes that the previously observed difference in Tyr levels between the expression of de-regulated ADHα and Tyr-sensitive ADHβ isoforms are not the consequence of localization to different sub-cellular compartments, with differing pools of available arogenate substrate.

**Figure 3:**
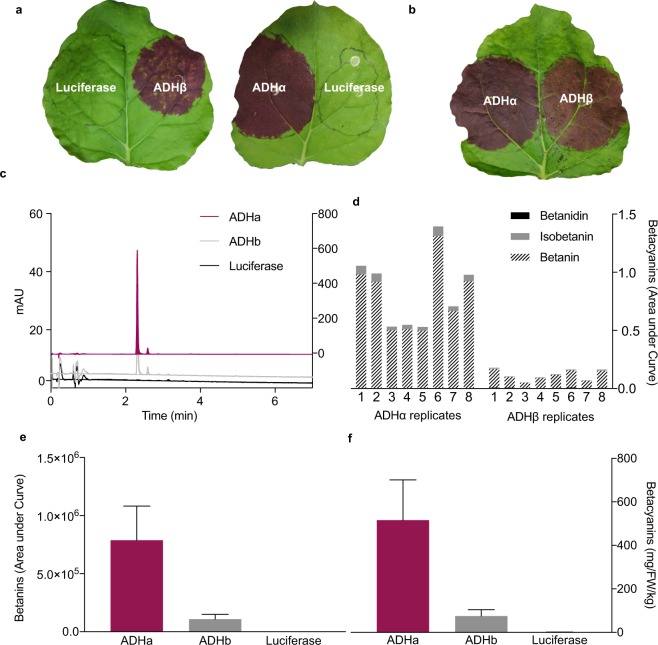
The relative efficacy of ADHα and ADHβ to boost production of Tyr-derived betalain metabolites. (**a**) Purple pigmentation induced by both *ADHα*-BET and *ADHβ*-BET vectors, relative to absent pigmentation in the *nos*::*Luciferase* control. (**b**) Relative pigmentation induced by *ADHα*-BET versus *ADHβ*-BET, after three days, indicating more intense pigmentation induced by *ADHα*-BET. (**c**) HPLC chromatograms for *ADHα*-BET, *ADHβ*-BET, *nos*::*Luciferase.* (**d)** Three main classes of betacyanins detected by the photodiode array: betanin, isobetanin, and betanidin for eight replicates of ADHα-BET versus ADHβ-BET (**e**) production of betanin, from eight replicates for ADHα-BET versus ADHβ-BET and *nos*::*Luciferase*. (**f**) Average mass of betalain produced from eight replicates of ADHα-BET versus ADHβ-BET and *nos*::*Luciferase*.

We then coupled the ADH isoforms to a Tyr-derived biosynthetic pathway producing betalains. Here, we isolated the three essential enzymes of the committed betalain pathway: *DODA and CYP76AD1* from *Beta vulgaris* and *cDOPA-5GT* from *Mirabilis jalapa*, and assembled these into a multi-gene vector following a previously published design (See Methods)^13–15^. Additionally, either *ADHα* or *ADHβ* from *Beta vulgaris* was incorporated. The firefly luciferase gene was also included to adjust for differences in transformation efficiency between replicates, and for within-leaf variation between infiltration sites (See Methods) (hereafter these multi-gene vectors are referred to as *ADHα*-BET and *ADHβ*-BET, Supplementary Fig. 1a,b). Transient expression of *ADHα*-BET versus *ADHβ*-BET in *N. benthamiana*, generated notable pigment production, three-days post- infiltration, which was absent in the luciferase control (Fig. 3a,b, Supplementary Fig. 1c). Incorporation of *ADHα* results in noticeably darker and more intense purple pigmentation spots relative to the *ADHβ* construct (Fig. 3b), suggesting enhanced production of Tyr-derived betalain pigments.

To confirm these qualitative observations, we further quantified the levels of betalains in two ways. First, liquid chromatography-mass spectrometry (LC-MS) analysis was conducted to identify and quantify the betacyanin pigments produced (Fig. 3c). Three main types of betacyanins, betanin, isobetanin, and betanidin (Fig. 3d), were identified with betanin comprising over 90% of total betacyanin observed (Supplementary Fig. 2a–e). We then quantified relative betalain content between *ADHα*-BET and *ADHβ*-BET infiltration spots, using the predominant compound, betanin, as a proxy for total betalain content. The incorporation of ADHα resulted in an average 7.3 fold increase in betanin production relative to the incorporation of ADHβ (Fig. 2e), indicating that about 70% of our previously described ten-fold increase in Tyr levels^8^ are successfully translated to the enhanced accumulation of downstream Tyr-derived metabolites. Second, the spectrophometric absorbance spectra for betacyanins was measured at 540 nm, corrected for the effect of chlorophyll *a* absorbance, to quantify the actual mass of betalains produced. Here our data indicate that inclusion of ADHα yields an average of 516 mg·kg^−1^·FW, relative to 74.40 mg·kg^−1^·FW with ADHβ, a similar 7-fold increase in betalain production (Fig. 2f). With an upper range of 855 mg·kg^−1^·FW, the ADHα enhanced yields of betalains compare favourably to many *in vitro* and commercial sources of betalain pigment^17^ emphasising the potential of manipulating primary metabolism to enhance the yield of specialised metabolites *in planta*. Finally, we confirmed that the relative efficacy of ADHα and ADHβ was not due to differences in transformation efficiency, by replicating these experiments normalised to luciferase expression (Supplementary Fig. 3a,b).

We have demonstrated the efficacy of the de-regulated ADHα to override intrinsic metabolic regulation of Tyr metabolism in a heterologous host plant, to enhance the over-production of the Tyr-derived metabolites, betalain pigments. In particular, our data emphasise the value of utilising naturally occurring de-regulated isoforms, in this case, ADHα, to re-engineer the interface between primary and specialised metabolic pathways^18^. The fact that the most common pathway for Tyr synthesis in plants is ADH-dependent, emphasises the broad utility of this de-regulated ADHα isoform for the enhanced production of Tyr-derived pharmaceuticals in diverse plant hosts^8^. It is notable that a considerable portion (>70%) of the elevated Tyr (relative to the elevated Tyr from ADHβ) is subsequently incorporated into the Tyr-derived betalain pigments, indicating efficient downstream processing in response to increased Tyr availability. However, in this instance, both the de-regulated ADHα and the betalain pathway, are peculiar to the plant order Caryophyllales^8,11^, and it is likely that the betalain pathway evolved in the context of, or in response to, enhanced Tyr metabolism^3^, ensuring that the reconstituted betalain pathway is predisposed to utilise elevated availability of the Tyr precursor.

Future work should explore the utility of relaxed ADH isoforms for other pharmaceutical production *in planta*. As demonstrated with the optimisation of Tyr-derived metabolic pathways in microbial platforms, additional rate-limiting steps may constrain the gains in Tyr-derived metabolites^10^ despite overall increases in Tyr availability. Also a further topic for evaluation is the broader consequence of overriding Tyr regulation for general plant host metabolism, which may limit carbon flow into phenylalanine-derived pathways^8^, and may affect production of physiologically significant compounds such as anthocyanins, flavonoids, and lignin^3^. Further characterisation of the key residues responsible for the de-regulated ADHα isoform, should allow refined gene-editing approaches *in planta*, to subtly moderate Tyr metabolic regulation yielding enhanced production of Tyr-derived metabolites, whilst limiting pleotropic physiological consequences.

## Methods

### Plant material and growth conditions

*Beta vulgaris ssp. vulgaris* ‘Bolivar’ (referred to as *B. vulgaris*) was obtained from Thompson & Morgan, Suffolk, UK. *Nicotiana benthamiana* is a standard laboratory line maintained by selfing. *B. vulgaris* plants were grown in the Cambridge University Botanic Gardens (CUBG) [under natural light and temperature conditions]. *N. benthamiana* plants were grown in soil under long day conditions (16 h light; 8 h dark) in controlled growth rooms maintained at 20 °C with 60% humidity.

### RNA extraction and cDNA synthesis

Tissue was snap frozen in liquid nitrogen and stored at −80 °C until needed. Frozen tissue was ground to a fine powder with a mortar and pestle in liquid nitrogen. RNA extraction was performed on 100 mg ground tissue using either Concert Plant RNA Reagent (Invitrogen, Carlsbad, CA, USA) followed by the TURBO DNA-free kit (Ambion, Carlsbad, CA, USA) to remove DNA, or the QIAGEN RNeasy Plant Mini Kit (Hilden, Germany) with DNA removed on-column using the QIAGEN RNase-Free DNase Set (Hilden, Germany), all according to the manufacturer’s specifications. RNA concentration was quantified by Nanodrop and RNA integrity was assessed by agarose gel electrophoresis. cDNA libraries were prepared using BioScript Reverse Transcriptase (Bioline Reagents, London, UK) and an oligo dT primer, according to the manufacturer’s recommendations.

### Gene isolation

*ADH*, *DODA* and *CYP76AD1* sequences were isolated from *B. vulgaris* leaf and/or hypocotyl cDNA libraries. *MjcDOPA-5GT* was isolated from red flowers of *M. jalapa*. Transcripts were amplified by PCR using Phusion High-Fidelity DNA polymerase (Thermo Fisher Scientific, Waltham, MA, USA) using the oligonucleotide primers listed in Supplementary Table 1. PCR products were ligated into the pBlueScript SK cloning vector using T4 DNA ligase (New England Biolabs, Hitchin, UK) and plasmids were sequenced to confirm gene identity. Sequencing services were provided by Source BioScience (Nottingham, UK) and retrieved sequences were analysed using *Geneious* software (Biomatters, Auckland, NZ).

### Transient expression assays in *Nicotiana benthamiana*

Transient gene expression assays in *N. benthamiana* were performed according to the previously described agroinfiltration method^19^ with some modifications. All constructs were transformed into the *Agrobacterium tumefaciens* GV3101 strain, and grown at 28 °C in LB media supplemented with antibiotics until reaching an OD_600_ of approximately 1.5. Cultures were then brought to a final OD_600_ of approximately 0.5 in infiltration media (10 mM MgCl2, 0.1 mM acetosyringone, 10 mM MES at pH 5.6). Cultures were left at room temperature for 2–3 h before infiltration. Infiltration was performed on 6-week old *N. benthamiana* plants and individual plants represented independent biological replicates. Young, expanding leaves were chosen to infiltrate and infiltration was facilitated by first generating a small nick on the adaxial leaf surface. The positioning of infiltration spots was alternated between biological replicates to account for intra-leaf variation^20^.

### Subcellular localisation of ADH

Subcellular localization of BvADHα and BvADHβ was assessed by C-terminal fusion of a Green Fluorescent Protein (GFP) and visualized using confocal fluorescence microscopy. Goldengate cloning was used to produce GFP-tagged BvADHα and BvADHβ by cloning via *Bpi*I into the pAGM1287 level 0 acceptor vector for CDS non-stop modules, and directly cloned into the pICH8988 level 2 binary vector via BsaI alongside the pICSL50016 GFP C-tag vector contained in the MoClo Plant Parts Kit (Addgene, Cambridge, MA, USA) to produce p35S:: BvADHα-GFP and p35S::BvADHβ-GFP. A control construct, p35S::GFP, containing only GFP was generated using the pICSL80005 level 0 vector from the MoClo Plant Parts Kit (Addgene, Cambridge, MA, USA). The pICH8988 level 2 expression vector contains a Cauliflower Mosaic Virus 35S (35S) promoter and *Agrobacterium tumefaciens*’ octopine synthase (ocs) terminator. The GFP gene contained in the pICSL50016 and pICSL80005 plasmids is a variant of turboGFP codon-optimised for plants from *Pontellina plumata* contained in the MoClo Plant Parts Kit (Addgene, Cambridge, MA, USA). Transient expression of 35S::BvADHα-GFP, 35S::BvADHβ-GFP and 35S::GFP was performed as described above. Three days post infiltration, leaf tissue from three biological replicates was observed under a Leica TCS SP8 X confocal microscope. GFP and chlorophyll were excited at 488 nm, and the emission was detected at 494–537 nm and 601–708 nm for GFP and chlorophyll, respectively. Images were further analysed using Fiji software^21^ and the Bio-Formats plugin^22^.

### Generation of vectors for betalain synthesis

The construction of multi-gene vectors containing the betalain biosynthetic genes was carried out using Golden Gate cloning^23,24^. Where necessary, gene sequences were domesticated to remove *Bsa*I and *Bpi*I restriction enzyme sites and alternative nucleotides were chosen to ensure no alteration to the amino acid sequence, using codon optimisation for *N. benthamiana*. Each multigene vector also included the firefly (*Photinus pyralis*) luciferase gene to adjust for differences in transformation efficiency and within leaf variation^20^. The luciferase gene was obtained from the plasmid pNWA62 provided by Dr Nick Albert (Plant and Food Research, Palmerston North, New Zealand) and had previously been modified to include an intron and codon optimised in order to enhance translation^25^. Cloning components were acquired from the MoClo Plant Tool Kit and the MoClo Plant Parts Kit (Addgene, Cambridge, MA, USA), except for the *A. thaliana* Ubiquitin 10 (Ubq10) promoter which was provided by Dr Nicola Patron (Earlham Institute, Norwich, UK). Cloning proceeded through Level 0, Level 1 and Level 2 modules using the long one-pot, one-step Type IIS mediated cloning reaction previously described^26^. Constitutively expressed promoters and terminators were used for all Level 1 transcriptional units: *PpLUC* under control of the *nos* promoter and terminator; *DODA* under control of the long *CaMV 35S* promoter and the *nos* terminator; CYP76AD1 under control of the long *CaMV 35S* promoter and the *A. thaliana* actin 2 (*act2)* terminator; *cDOPA5GT* and *ADH* under control of the *Ub10* promoter and *35S* terminator. Level 2 binary vectors were comprised of transcriptional units in the following positional order: *PpLUC* (reverse orientation); *DODA; CYP76AD; cDOPA5GT; ADH*. Level 0 and Level 1 vectors were confirmed by sequencing of the full inserts. Level 2 vectors were confirmed by sequencing the boundaries of the inserts and the gene variant, and performing diagnostic restriction enzyme digests.

### Betalain quantification using LC-MS

For each infiltration spot, 25–40 mg of fresh weight leaf tissue was sampled and snap frozen in liquid nitrogen in 2 ml tubes with one 5 mm glass bead. Sampled leaf tissue was ground frozen using a Tissue Lyser II homogeniser (QIAgen, Hilden, Germany). Betalains were extracted overnight at 4 °C in 80% aqueous methanol with 50 mM ascorbic acid with a volume of 1 mL extraction buffer per 50 mg leaf tissue. After extraction, the samples were clarified by centrifuging at 21,130 g for 10 mins and collecting the supernatants. For each sample, 250 uL of supernatant was dried *in vacuo* using a SpeedVac and sent to the Molecular Analysis Service at the John Innes Centre (JIC; Norwich, UK) for analysis. Samples were resuspended in water before analysis. Betalains were analysed on a Nexera/Prominence UHPLC system equipped with a PDA detector and an ion-trap time-of-flight (IT-ToF) mass spectrometer (Shimadzu, Kyoto, Japan). Separation was on a 100 × 2.1 mm 2.6 μ Kinetex EVO C18 column (Phenomenex) using the following gradient of acetonitrile (B) versus 1% formic acid in water (A), run at 40 °C and 400 μL.min^−1^: 0 min, 2% B; 2 min, 10% B; 7 min, 30% B; 9 min, 90% B, 9.8 min, 90% B; 9.85 min, 2% B; 13 min, 2% B. Between 9.85 min and 12.95 min the flow rate was increased to 550 μL.min^−1^. Betalains were detected by UV/vis absorbance and positive electrospray MS. The PDA collected spectra from 200–650 nm at 6.25 Hz with a time-constant of 0.08 sec. A beetroot hypocotyl sample was included as a control in order to identify known betalain compounds using retention time and spectral maxima, which could be further verified by mass-spec analysis. The MS collected full spectra from *m/z* 200–2000 and data-dependent MS2 (*m/z* 50–2000) of the most abundant precursor ions, at an isolation width of *m/z* 3.0, 50% collision energy and 50% collision gas. Spray chamber conditions were 250 °C curved desorbation line, 1.5 L.min^−1^ nebulizer gas, and 300 °C heat block temperature with drying gas “on”. The instrument was calibrated using sodium trifluoroacetate cluster ions according to the manufacturer’s instructions, immediately before analysis. To quantify relative betalain content between ADHα and ADHβ infiltration spots, the predominant betalain component, betanin, was chosen as a proxy for total betalain content.

### Total mass quantification of betalains using spectrophotometer

Betalain content was estimated spectrophotometrically using a SANYO SP75 UV-VIS (Sanyo, Osaka, Japan) spectrophotometer, as _*A538* − (*0.46* × *A662*)_, where A538 and A662 are the absorbance values for betacyanins and chlorophyll *a* at 538 nm and 662 nm respectively. The subtraction of _(*0.46* × *A662*)_ compensated for the small overlap in absorption by extracted chlorophyll and the correction factor was recalculated for this extraction method. Absorbance values were converted to betanin equivalents using the molar extinction coefficient ε = 60 000 l mol^−1^ cm^−1^ and molecular weight = 550 g-mol^27^.

### Betalain quantification using plate reader to normalise for luciferase

Leaf tissue was sampled three days post-infiltration with a leaf corer (9 mm diameter) and snap frozen in liquid nitrogen in 2 ml tubes with two 3 mm glass beads. Five technical replicates were sampled for each infiltration spot. Sampled leaf tissue was ground frozen using a Tissue Lyser II homogeniser (QIAgen, Hilden, Germany). Homogenised samples were resuspended in 300 μl of SPB extraction buffer (50 mM sodium phosphate buffer, 2 mM dithiothreitol, 10% v/v glycerol, 1% v/v Triton X-100)^28^ and mixed by vortexing. Samples were then centrifuged at 12,100 g for 10 min, and 100 μl of each supernatant was transferred to individual wells of a black µCLEAR 96-well microplate (Greiner Bio-One, Kremsmünster, Austria). Luminescence and absorbance were measured for each well with a CLARIOstar microplate reader (BMG Labtech, Aylesbury, UK). To measure luminescence, 100 μl of SteadyGlo Luciferase Assay Substrate (Promega, Madison, WI, US) was added to each well before plate reading and luminescence was measured at 25 °C using standard settings with no filter. To ensure that luminescence levels fell within a linear range, a luciferase standard curve was also produced using five 1:10 serial dilutions of QuantiLum Recombinant Luciferase (Promega, Madison, WI, US) in SPB supplemented with 1 mM bovine serum albumin (BSA). Absorbance values ranging from 400–700 nm were measured with a resolution of 5 nm. Betacyanin relative concentration was calculated as *A*540 − (0.1 × *A*660) where A540 and A660 are the absorbance values for betacyanins and chlorophyll *a* at 540 nm and 660 nm respectively, corrected to the average SPB buffer values for each wavelength. Obtained betacyanin values were then normalised to the luciferase luminescence measured for each well and corrected to the average luminescence units recorded for the SPB buffer alone.

## Electronic supplementary material

Combined Supplementary Figures and Table

## Data Availability

The datasets generated during and/or analysed during the current study are available from the corresponding author on reasonable request. Sequences are deposited in GenBank under the following accession numbers: MH836616, MH836617, MH836618.
