# Supplementary material for: Redirecting Primary Metabolism to Boost Production of Tyrosine-Derived Specialised Metabolites in Planta
Source: Sci Rep. 2018 Nov 22;8:17256. doi: 10.1038/s41598-018-33742-y (PMC6250739; doi:10.1038/s41598-018-33742-y)
Supplement: Supplementary file 1 — Combined Supplementary Figures and Table [file 41598_2018_33742_MOESM1_ESM.pdf]

SUPPLEMENTARY INFORMATION TITLE PAGE

REDIRECTING PRIMARY METABOLISM TO BOOST PRODUCTION OF TYROSINE-DERIVED SPECIALISED METABOLITES *IN PLANTA*

Alfonso Timoneda[1]*, Hester Sheehan[1]*, Tao Feng[1], Samuel Lopez-Nieves[2], Hiroshi Maeda[2], Samuel Brockington[1]

SUPPLEMENTARY INFORMATION LEGENDS

Supplementary Figure 1: Vector Maps: a, ADHα-BET; **b** ADHβ-BET; **c,** *nos*::Luciferase

Supplementary Figure 2. LC-MS data for detected betalain constituents: a, the relative percentage of betacyanins contributed by three main classes of detected pigments – betanidins, isobetanin, and betanin; MS spectra confirming identity of component betacyanins: **b,** full LCMS spectrum for beetroot hypocotyl sample; **c**, MS2 for the precursor ion 551.15; **d,** full LCMS spectrum for representative *ADHα*-BET sample; **e**, MS2 for the precursor ion 551.15

Supplementary Figure 3. Absorbance measurement for *ADHα*-BET versus *ADHβ*-BET normalized to luciferase expression. **a**, Absorbance of *ADHα*-BET versus *ADHβ*-BET for eight independent leaf replicates as measured by absorbance at 540nm, corrected for a chlorophyll *a* absorbance and normalized by luciferase expression; **b,** average and standard deviation of pigment absorption across all eight replicates for *ADHα*-BET versus *ADHβ*-BET. Error bars represent the standard deviation calculated for five technical replicates.

Supplementary Table 1. Oligonucleotides used to isolate betalain pathway genes for vector constructs.

Supplementary Figure 1

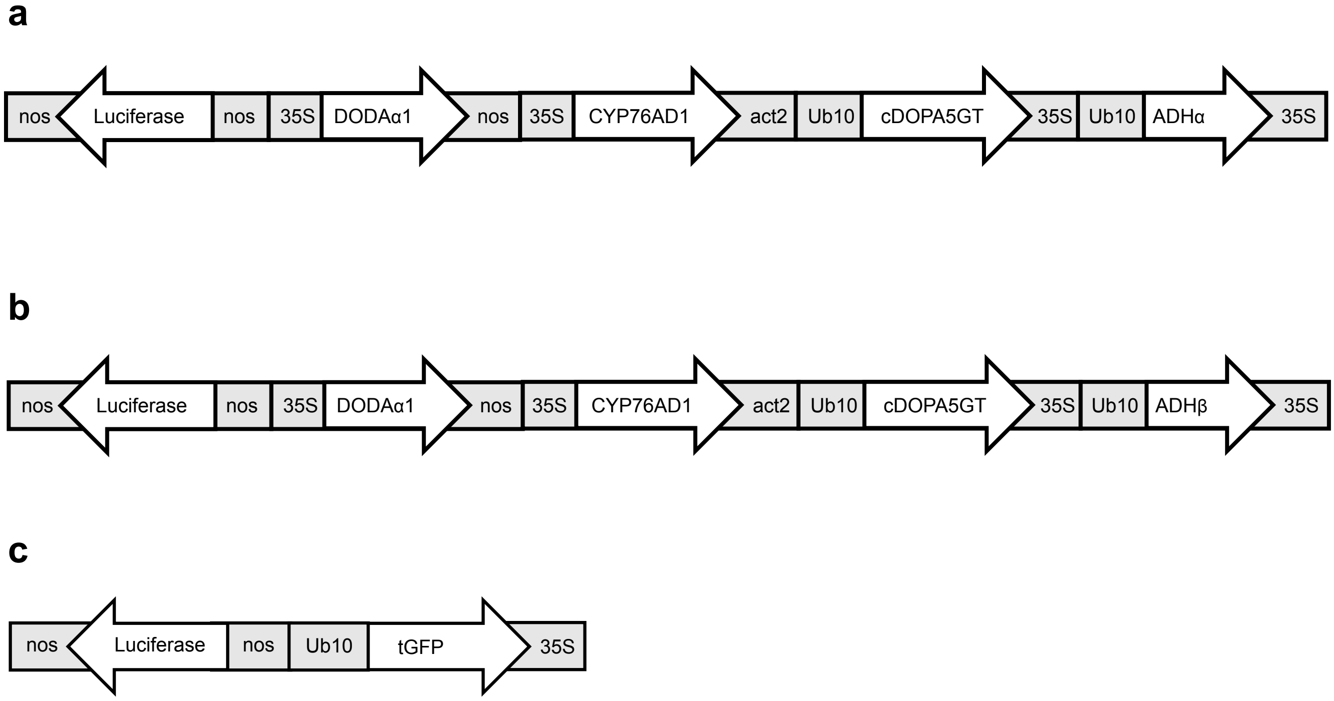

Supplementary Figure 2

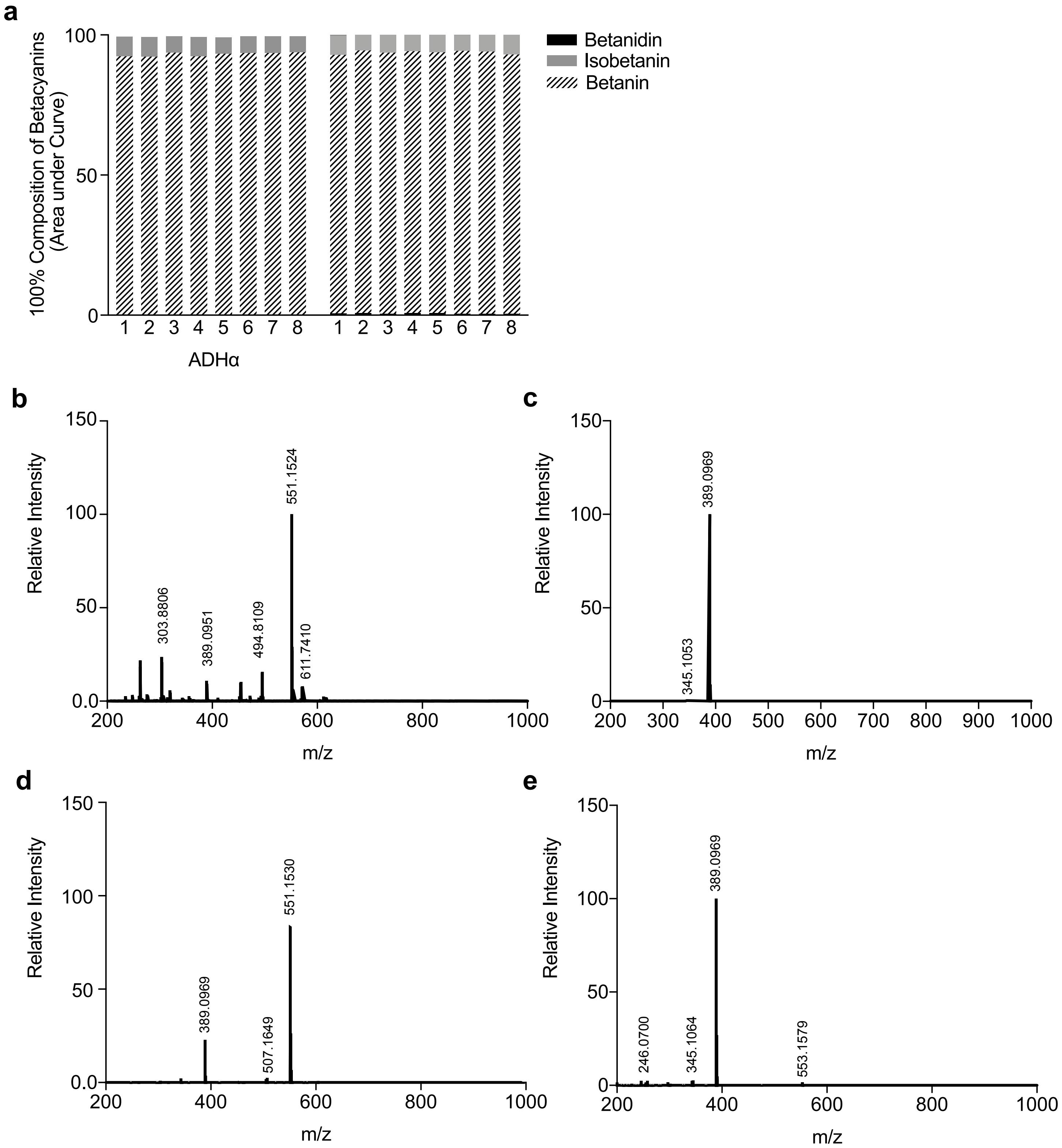

Supplementary Figure 3

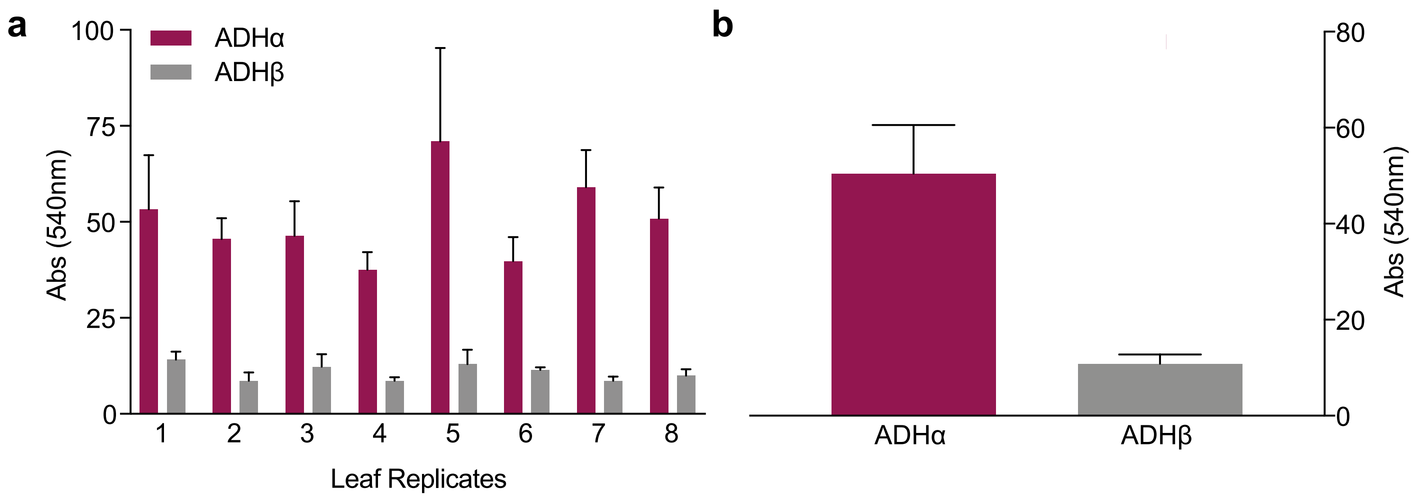

Supplementary Table 1

ID	Sequence 5'-3'
BvADHa_F	ATGATTTCACTCTCTTCTTTTCATCC
BvADHa_R	CTATCTAGTACCATTAACCACCACTAAATC
BvADHb_F	ATGCTTTCTCTCTCCTCCAC
BvADHb_R	TCAATTTGTCTCCGAATTTG
BvDODA_a1_F	ATGAAAATGATGAATGGAGAAG
BvDODA_a1_R	CTAGGCTGAAGTGAACTTGTAG
BvCYP76AD1_F	ATGGATCATGCAACATTAGCA
BvCYP76AD1_R	TCAATACCTAGGTATTGGAATAAGTTTTAA
MjcDOPA5GT_F	ATGACCGCCATTAAAATG
MjcDOPA5GT_R	TTATTGAAGAGAAGGTTCCAAC
